# Draft Genome Sequence of *Xylella fastidiosa* Pear Leaf Scorch Strain in Taiwan

**DOI:** 10.1128/genomeA.00166-14

**Published:** 2014-03-20

**Authors:** C.-C. Su, W.-L. Deng, F.-J. Jan, C.-J. Chang, H. Huang, J. Chen

**Affiliations:** a Taiwan Agricultural Chemicals and Toxic Substances Research Institute, Wufeng, Taichung, Taiwan; b Department of Plant Pathology, National Chung Hsing University, Taichung, Taiwan; c Department of Plant Pathology, University of Georgia, Griffin, Georgia, USA; d School of Information, University of South Florida, Tampa, Florida, USA; e U.S. Department of Agriculture, Agricultural Research Service, San Joaquín Valley Agricultural Sciences Center, Parlier, California, USA

## Abstract

The draft genome sequence of *Xylella fastidiosa* pear leaf scorch strain PLS229, isolated from the pear cultivar Hengshan (*Pyrus pyrifolia*) in Taiwan, is reported here. The bacterium has a genome size of 2,733,013 bp, with a G+C content of 53.1%. The PLS229 genome was annotated and has 3,259 open reading frames and 50 RNA genes.

## GENOME ANNOUNCEMENT

*Xylella fastidiosa*, a Gram-negative bacterium, causes pear leaf scorch (PLS) disease in Taiwan (1). The disease was observed around 1991 in the area where the low-chilling pear cultivar Hengshan (*Pyrus pyrifolia*) was grown. The pathogen resides in the xylem tissues of the host plant and can be transmitted through grafting. The PLS strain is one of the few *X. fastidiosa* strains reported outside the Americas (1–3). Early serological tests indicated that the PLS strain is unique compared to other known *X. fastidiosa* strains (1). Analyses using DNA-DNA hybridization (4), sequences of 16S rRNA genes and 16S-23S rRNA intergenic transcribed sequences (16S-23S ITS) (5), sequences from 18 genomic loci (6), and randomly amplified polymorphic DNA (RAPD) profiles (7) further confirmed that the PLS strain is distinct from the currently known subspecies, i.e., *X. fastidiosa* subsp. *fastidiosa*, *X. fastidiosa* subsp. *multiplex*, and *X. fastidiosa* subsp. *pauca* (8).

*X. fastidiosa* is nutritionally fastidious (9). Characterization of the bacterium through the traditional cultivation-based methodologies has yielded limited information. Whole-genome sequencing has been an efficient technique for generating biological information for fastidious prokaryotes. Both complete and shotgun whole sequences of the known subspecies of *X. fastidiosa* have been published (10–17). Here, we report a draft whole-genome sequence of an *X. fastidiosa* PLS strain.

*X. fastidiosa* strain PLS229 was isolated from the symptomatic pear cultivar Hengshan (*Pyrus pyrifoliae*) at Houli (24°20′14″N, 120°44′11″E), Taiwan, and triple-cloned. To obtain genomic DNA, strain PLS229 was cultured in PD2 broth (18) at 28°C for 5 to 6 days. The bacterial cells were collected by centrifugation. Total genomic DNA was extracted according to a procedure described previously (5). Genome sequencing was carried out on a 454 GS-FLX system using Titanium chemistry (Roche) (19). Paired-end reads were assembled with the Newbler software (version 2.6; Roche Diagnostics). The PLS229 genome consists of 2,733,013 bp (~20× coverage; G+C content, 53.1%) assembled into 85 contigs ranging from 525 bp to 230,364 bp. Annotation was performed by the RAST server (http://rast.nmpdr.org/) (20), which utilized GeneMark, Glimmer, and tRNAscan-SE searches. The PLS229 genome was predicted to have a total of 3,259 open reading frames (ORFs) and 50 RNA genes.

The sequences of *ssr* (16S rRNA) and three housekeeping genes, *gyrB* (DNA gyrase subunit B), *dnaK* (chaperone protein), and *rpoD* (RNA polymerase sigma factor), were selected and compared, using BLAST analyses (21), to the corresponding gene sequences of *X. fastidiosa* strains deposited in GenBank. The *ssr* sequence shares 100% similarity to that of the previously published *X. fastidiosa* PLS strain (5) and 99% similarity to those of *X. fastidiosa* subsp. *fastidiosa* (strains Temecula1 and M23), *X. fastidiosa* subsp. *multiplex* (strain M12), and *X. fastidiosa* subsp. *pauca* (strain 9a5c). All these further confirmed that the sequenced bacterial strain is a member of *X. fastidiosa*. Strain PLS229 is similar to all three subspecies of *X. fastidiosa* (strains 9a5c, M12, M23, and Temecula1), with 88% at locus *gyrB*, 89% at locus *dnaK*, and 88% at locus *rpoD*, suggesting the uniqueness of strain PLS229.

### Nucleotide sequence accession numbers.

This whole-genome shotgun project has been deposited at DDBJ/EMBL/GenBank under the accession no. JDSQ00000000. The version described in this paper is version JDSQ01000000.
